# Emotional Response Inhibition Is Greater in Older Than Younger Adults

**DOI:** 10.3389/fpsyg.2019.00961

**Published:** 2019-05-01

**Authors:** Jill D. Waring, Taylor R. Greif, Eric J. Lenze

**Affiliations:** ^1^Department of Psychology, Saint Louis University, St. Louis, MO, United States; ^2^Department of Psychiatry, Washington University School of Medicine, St. Louis, MO, United States

**Keywords:** aging, emotion, response inhibition, executive function, emotion regulation, go/no-go, older adults

## Abstract

Emotional information rapidly captures our attention and also often invokes automatic response tendencies, whereby positive information motivates approach, while negative information encourages avoidance. However, many circumstances require the need to override or inhibit these automatic responses. Control over responses to emotional information remains largely intact in late life, in spite of age-related declines in cognitive control and inhibition of responses to non-emotional information. The goal of this behavioral study was to understand how the aging process influences emotional response inhibition for positive and negative information in older adults. We examined emotional response inhibition in 36 healthy older adults (ages 60–89) and 44 younger adults (ages 18–22) using an emotional Go/No-Go task presenting happy (positive), fearful (negative), and neutral faces. In both younger and older adults, happy faces produced more approach-related behavior (i.e., fewer misses), while fearful faces produced more avoidance-related behavior, in keeping with theories of approach/avoidance-motivated responses. Calculation of speed/accuracy trade-offs between response times and false alarm rates revealed that younger and older adults both favored speed at the expense of accuracy, most robustly within blocks with fearful faces. However, there was no indication that the strength of the speed/accuracy trade-off differed between younger and older adults. The key finding was that although younger adults were faster to respond to all types of faces, older adults had greater emotional response inhibition (i.e., fewer false alarms). Moreover, younger adults were particularly prone to false alarms for happy faces. This is the first study to directly test effects of aging on emotional response inhibition. Complementing previous literature in the domains of attention and memory, these results provide new evidence that in the domain of response inhibition older adults may more effectively employ emotion regulatory ability, albeit on a slower time course, compared to younger adults. Older adults’ enhanced adaptive emotion regulation strategies may facilitate resistance to emotional distraction. The present study extends the literature of emotional response inhibition in younger adulthood into late life, and in doing so further elucidates how cognitive aging interacts with affective control processes.

## Introduction

Emotion has a robust and enduring influence on behaviors across many domains of cognition and perception. Emotional information rapidly captures our attention, receives priority in mental processing, and invokes automatic or habitual responses in a variety of contexts (Pessoa and Ungerleider, 2004). For example, emotional valence often biases response tendencies, whereby positive information motivates approach, while negative information encourages avoidance (Elliot, 2006; Phaf et al., 2014). However, those response biases are often either undesirable or contextually inappropriate, and in such circumstances, executive control – or regulation – over one’s emotionally driven behavior is required to implement more desirable or adaptive responses (Banich et al., 2009). One direct “real world” application of unsuccessful response inhibition could be in instances where one is aware of their desired or most situationally appropriate reaction, yet cannot override an automatic or habitual response tendency. For example, failure to suppress outbursts of anger or frustration at times when holding your tongue would be the more optimal reaction.

Flexible and effective adaptation of behavior to context is an important facet of executive functioning, and hinges upon effective inhibition and resistance to distractions (Diamond, 2013). However, the normal aging process substantially diminishes performance in many cognitive domains (Park et al., 2002), including cognitive control and inhibition abilities (Hasher and Zacks, 1988; West and Alain, 2000; Gazzaley and D’Esposito, 2007; Gazzaley et al., 2008; Sebastian et al., 2013). Critically though, in contrast to age-related declines in cognitive control over responses to non-emotional information, control over responses to emotional information (i.e., emotion regulation) remains intact or is even enhanced in late life (reviewed by Kryla-Lighthall and Mather, 2009; Samanez-Larkin et al., 2009; Zinchenko et al., 2017). One example of intact emotion regulation in older adults is the facilitated attention and better memory for positive information compared to negative, which has been termed the “Positivity Effect” (Charles et al., 2003; Mather and Carstensen, 2005; Carstensen et al., 2006). Positivity effect theorizes that older adults implement chronically active emotional regulation goals to prioritize attentional focus and processing resources toward positive information, in contrast to younger adults who do not have such chronically active goals (Kryla-Lighthall and Mather, 2009; Reed and Carstensen, 2012). However, the positivity effect is nuanced and does not consistently arise within all domains of cognition and perception. The strength of the effect also varies with task demands. For instance, the positivity effect in memory is attenuated or eliminated in tasks that explicitly instruct participants attend to positive and negative stimuli or that impose high cognitive demands (Mather and Knight, 2005; Samanez-Larkin et al., 2009; Brassen et al., 2011; Reed et al., 2014). Moreover, there is little evidence that the positivity effect differentially affects basic abilities to label positive vs. negative stimuli (Ruffman et al., 2008). Thus, although older adults have well preserved emotion regulation abilities, which often prioritize positive over negative experiences and information processing, a positivity bias is not evident in all cognitive and perceptual tasks.

The contrast between declining cognitive control and enhanced emotion regulation in late life suggests an intriguing divergence of cognitive and affective processing in older adults. However, this topic has received little research attention. The dearth of literature in older adults contrasts with the large and growing literature on this topic from childhood through younger adulthood. The development of inhibitory control over responses to affective information (and requisite prefrontal brain regions) during adolescence and emerging adulthood has received considerable academic and popular attention (Arnett, 2000; Tottenham et al., 2011; Cohen-Gilbert et al., 2014; Cohen et al., 2016), in recognition that the late teens and early twenties are a pivotal development period for these abilities. Developmental studies of response inhibition in affective contexts have reflected age-related increases in response inhibition from childhood, through adolescence, and into emerging adulthood (Schulz et al., 2007; Schel and Crone, 2013; Cohen-Gilbert et al., 2014). Mounting evidence suggests that better behavioral response inhibition to emotional faces corresponds with development of prefrontal brain regions (Todd et al., 2012; Cohen et al., 2016), highlighting that the prefrontal cortex plays an important role in determining and executing appropriate responses to emotional stimuli. The developmental improvement of emotional response inhibition in particular may be greater than improvements in non-emotional response inhibition (Tottenham et al., 2011). Evidence from several studies with younger adults show that approach/avoidance motivations for positive and negative information, respectively, manifest as poorer emotional response inhibition for positive than negative stimuli (Hare et al., 2005; Schulz et al., 2007; Chiu et al., 2008; Tottenham et al., 2011). However, it is an open question how approach/avoidance motivations could interact with the demands of an emotional response inhibition task in older adults.

Older adults’ inhibition of responses to emotional information is presently an unexamined area of research. To date there has been some investigation of the impact of aging on other related domains of executive functioning, but none that has specifically set out to investigate older adults’ ability to stop (i.e., inhibit) a motor response to emotional stimuli. “Emotional Stroop” (Wurm et al., 2004; LaMonica, 2010) and “emotion conflict” tasks (Monti et al., 2010; Zinchenko et al., 2017) assess ability to monitor and select among competing or conflicting attributes within a stimulus (i.e., within trial). In emotional Stroop and emotion conflict tasks individuals must select among task-relevant and task-irrelevant stimulus attributes within each trial, in accordance with task instructions (Etkin et al., 2006); the choice there is not whether to respond, but rather which response option to select. While one study did employ a task of response inhibition for happy and angry faces in an older adult sample (e.g., go/no-go task), the authors assessed only accuracy of responses to targets (i.e., “go” trials), but did not report results of response inhibition for non-targets (i.e., “no-go” trials), leaving persisting questions about how aging impacts emotional response inhibition (Bailey et al., 2009). Age-related changes in response inhibition especially for emotional information is an important area of investigation because there are well documented age-related changes in processing biases toward positive information and away from negative information in late life, in the face of overall declining executive function over neutral information (Gazzaley and D’Esposito, 2007; Reed et al., 2014). Thus, although prior research has characterized a rising curve of emotional response inhibition effectiveness from childhood through adolescence and into younger adulthood, it remains to be determined whether emotional response inhibition continues to improve into late life, or may decline in conjunction with cognitive control over non-emotional information.

The goal of the present study was to understand how the aging process influences emotional response inhibition in older adults by examining older adults’ ability to stop their responses to positive, negative, and neutral facial expressions, in comparison to younger adults. First, the strong evidence for improved emotional control (regulation) in older vs. younger adults suggests that emotional response inhibition should improve into older adulthood, and be spared from age-related decrements observed in cognitive (non-emotional) domains. Accordingly, we tested the hypothesis that response inhibition for emotional vs. non-emotional stimuli would differ between older compared to younger adulthood, where older adults would show relative disadvantage for resisting neutral non-targets, but relative advantage for resisting emotional non-targets. Secondly, informed by evidence that approach/avoidance motivational goals are often observed in younger adults (Hare et al., 2005; Schulz et al., 2007; Chiu et al., 2008; Tottenham et al., 2011), and also conform to the direction of age-related positivity biases (i.e., approach positive stimuli, avoid negative), we would not expect reduction in approach/avoidance-motivated behaviors from younger to older adulthood. As such, we hypothesized that response patterns (both of younger and older adults) would indicate differences between positive vs. negative stimuli, namely that there would be more false alarms for emotionally positive compared to negative non-targets, and also faster responses and fewer misses for positive vs. negative targets. Together, tests of these hypotheses elucidate how emotion regulatory abilities and approach/avoidance motivations shape patterns of emotional response inhibition from younger to older adulthood.

To test these hypotheses, the present study examined older and younger adults’ responses during a Go/No-Go task featuring emotional faces. We contrasted response patterns for target and non-target trials each containing an image of a positive, negative, or neutral facial expression. In this design, the Go/No-Go task allows assessment of discrimination (differentiation) between emotional and neutral faces, the impact of approach/avoidance motivations on response patterns, and how aging impacts each. The outcome measures assessed rate of missed responses to targets (emotion discrimination), rate of false alarms to non-targets (response inhibition), and response times to targets.

## Materials and Methods

### Participants

Forty-eight younger adults were recruited from the student populations of Saint Louis University and Santa Clara University who were taking psychology courses (22 female, 26 male; age: *M* = 19.04, *SD* = 1.20, range 18–22; yrs edu: *M* = 12.73, *SD* = 1.03, range 12–15). From this total, data from 4 individuals were excluded for not following task instructions [e.g., 3 people conflated go and no-go instruction in one block, and 1 person responded for 100% of trials in one block (i.e., 100% false alarms)]. The final sample included 44 younger adults (20 female, 24 male; age: *M* = 19.00, *SD* = 1.14, range 18–22; yrs edu: *M* = 12.70, *SD* = 1.00, range 12–15). Different analyses on a portion of the data for 39 of the younger adults are reported in Greif and Waring (2018). 39 older adults were recruited from the St. Louis community (20 female, 19 male; age: *M* = 70.18, *SD* = 7.39, range 60–89; yrs edu: *M* = 16.00, *SD* = 2.21, range 12–20). From this total, data from 3 were excluded: 1 for giving no responses in one task block, and 2 due to experimenter error (participant given incorrect task instructions). The final sample included 36 older adults (19 female, 17 male; age: *M* = 70.42, *SD* = 7.32, range 60–89; yrs edu: *M* = 15.92, *SD* = 2.27, range 12–20). Older adults were cognitively non-impaired, as operationalized by scores on the Mini Mental Status Exam (MMSE; Folstein et al., 1975) within normal range (*M* = 29.17, *SD* = 1.06, range 26–30). Sample race and ethnicity are reported in Supplementary Materials.

Study exclusion criteria included uncorrected vision or hearing problems, present or prior diagnosis or treatment of any psychiatric conditions, Autism Spectrum Disorder or Asperger’s Syndrome, colorblindness, history of stroke or severe head injury, or history of alcoholism or substance abuse within 6 months. Additional exclusion criteria for older adults included life-shortening illness (e.g., cancer), dementia, clinical neurodegenerative illness (e.g., Parkinson’s disease, cerebrovascular disease), or current use of any central nervous system (CNS)-altering medication, which included psychotropic medications as well as any other medications with CNS effects (e.g., centrally acting anticholinergics and antihistaminergics, opioids, GABAergic, and dopaminergics). Younger adults’ inclusion criterion was age 18–30. Older adults’ inclusion criteria were age 60 or older and ability to receive payment for work in the United States.

Desired sample size was determined *a priori* to assure that the study was sufficiently powered to detect interaction effects in the primary analyses, i.e., mixed within-between repeated measures ANOVA with 2 groups (e.g., factor “age group”) and 4 measurements (e.g., factor “block”). Power analyses calculated based upon a conservative effect size (*f* = 0.20), 0.01 alpha error probability (2-tailed), and 90% power determined a required total sample size of *n* = 64 (calculated using G^∗^Power 3.1). The present sample of *n* = 80 analyzed surpasses the size needed to attain sufficient statistical power in this design, minimizing possibility that the sample size was under-powered to detect effects.

### Procedures

The protocol was approved by the Institutional Review Boards of Saint Louis University, Santa Clara University, and Washington University in St Louis. Data were collected by the PI (JW) as well as trained graduate (TG) and undergraduate students. The training procedure included students observing the PI administer the protocol several times, followed by the PI shadowing the student administering the protocol, with supplemental detailed instruction in proper administration and scoring of neuropsychological measures.

All participants provided written informed consent in accordance with the Declaration of Helsinki. Participants at Saint Louis University and Washington University School of Medicine additionally provided HIPAA authorization. Younger adult participants were recruited and anonymously screened through online participant management systems [Sona Systems (Tallinn, Estonia) and Qualtrics (Provo, Utah), respectively]. If they did not meet any exclusion criteria (as described in “Participant” section above), they were given a code to sign up for a lab research appointment. Older adults who responded to community advertisements were screened over the phone for study inclusion and exclusion criteria. If they met eligibility criteria they were scheduled for a lab research appointment. After providing informed consent, all participants provided demographic information and again completed screening for eligibility criteria. Participants then completed the experimental task, as well as self-report measures and neuropsychological measures of cognition (described below in “Measures” section). It took 60–75 min to complete these procedures. Younger adults received course research participation credits, and older adults were paid for their time.

### Measures

Participants completed several neuropsychological measures to characterize age differences in cognition among participants, and to confirm the older adult sample was cognitively normal. Although not part of our primary aims, collecting neuropsychological measures also permitted exploratory comparisons between measures of cognition in non-emotional domains and the emotional Go/No-go task performance. Measures of cognition included the Trail Making Task parts A and B (Reitan, 1958), and Color-Word Interference (4 conditions) and Verbal Fluency Tasks (4 conditions) from the Delis-Kaplan Executive Functioning System (D-KEFS; Delis et al., 2001). In light of documented age differences in mood and anxiety (Machado et al., 2018) and in use of emotion regulation strategies (Urry and Gross, 2010), participants also completed self-report measures including the Emotion Regulation Questionnaire (ERQ; Gross and John, 2003), Spielberger State-Trait Anxiety Inventory (STAI; Spielberger and Gorsuch, 1983), and a depression inventory [older adults: Geriatric Depression Scale 30-item version (GDS; Yesavage et al., 1983); younger adults: Beck Depression Inventory (BDI; Beck et al., 1961)] to explore the possibility of differing relationships between these measures and emotional response inhibition as a function of aging. Following from our and others’ prior findings in younger adult samples (Mogg and Bradley, 2005; Pacheco-Unguetti et al., 2012; Greif and Waring, 2018), we explored whether higher anxiety or depression corresponded with poorer task performance (slower responses, more false alarms). Some measures were not administered to a small group of pilot participants during protocol development period (Trail Making Test: 2 younger adults, 4 older adults; ERQ and GDS: 1 older adult each). There were sporadic instances of missing data for the D-KEFS Color-Word Interference task and Trail Making Test due to participant and researcher errors during test administration (see Supplementary Methods for detail; sample sizes for each measure are reported in Table 1).

**Table 1:** Results of cognitive and self-report measures.

	Group	*N*	*M*	*SD*	Range	*F*	*P*
D-KEFS Color Word Interference							
Color naming (sec)	YA	43	25.91	4.01	18.56 – 35.75	27.43	<0.0005
	OA	36	33.04	7.79	21.69 – 57.22		
Word reading (sec)	YA	43	19.62	3.30	14.58 – 28.34	20.55	<0.0005
	OA	36	23.57	4.44	16.31 – 36.38		
Inhibition (sec)	YA	44	43.40	8.37	26.62 – 66.06	66.31	<0.0005
	OA	35	63.56	13.49	44.00 – 95.91		
Inhibition/switching (sec)	YA	20	49.01	7.03	33.82 – 65.81	14.79	<0.0005
	OA	34	70.63	24.48	42.06 – 180.00		
D-KEFS Verbal Fluency							
Letter fluency: FAS	YA	44	41.84	10.27	25 – 69	0.05	0.83
	OA	36	41.33	11.07	24 – 69		
Category fluency: Animals + Boys’	YA	44	43.18	7.31	32 – 60	3.92	0.05
Names	OA	36	39.72	8.30	23 – 60		
Category Switching: Fruits/Furniture	YA	44	14.61	2.88	10 – 24	7.81	0.01
	OA	36	12.72	3.17	4 – 19		
Trail Making Test							
Part A (sec)	YA	42	22.57	6.42	13.00 – 37.28	31.17	<0.0005
	OA	32	33.12	9.81	17.37 – 64.46		
Part B (sec)	YA	41	54.86	17.52	27.28 – 120.00	15.33	<0.0005
	OA	31	96.59	65.34	51.59 – 370.90		
ERQ reappraisal (avg)	YA	44	3.91	0.55	2.33 – 5.00	31.83	<0.0005
	OA	35	4.87	0.95	3.14 – 6.83		
ERQ suppression (avg)	YA	44	2.89	0.97	1.00 – 5.00	0.29	0.59
	OA	35	3.01	0.90	1.25 – 5.00		
STAI: state	YA	44	37.61	9.79	21 – 59	17.71	<0.0005
	OA	36	28.83	8.62	14 – 51		
STAI: trait	YA	44	41.45	8.36	23 – 64	40.89	<0.0005
	OA	36	30.11	7.28	10 – 44		
Geriatric Depression Scale	OA only	35	3.43	3.22	0 – 13	–	–
Beck Depression Inventory	YA only	44	7.43	5.88	0 – 24	–	–

*The D-KEFS Color Word Interference task inhibition/switching condition was not collected on participants at Santa Clara University due to experimenter error. Citations and description of sporadic missing values available in text. YA, younger adults; OA, older adults.*

### Stimuli

The NimStim Set of facial expressions were used as task stimuli (Tottenham et al., 2009^1^). Thirty-six grayscale faces depicted neutral, happy, and fearful expressions (12 each). Models were between age 21 to 30 years old (Tottenham et al., 2009). The image set contained a total of 12 male and female African-American, Asian, and Caucasian identities, and all 12 model identities were shown within each task block (more information about the stimulus set is available in Supplementary Materials). All images were normalized for size (approximately 9 cm in width by 12 cm in height) and luminance, and presented in the center of the screen against a black background.

### Experimental Task

**FIGURE 1:**
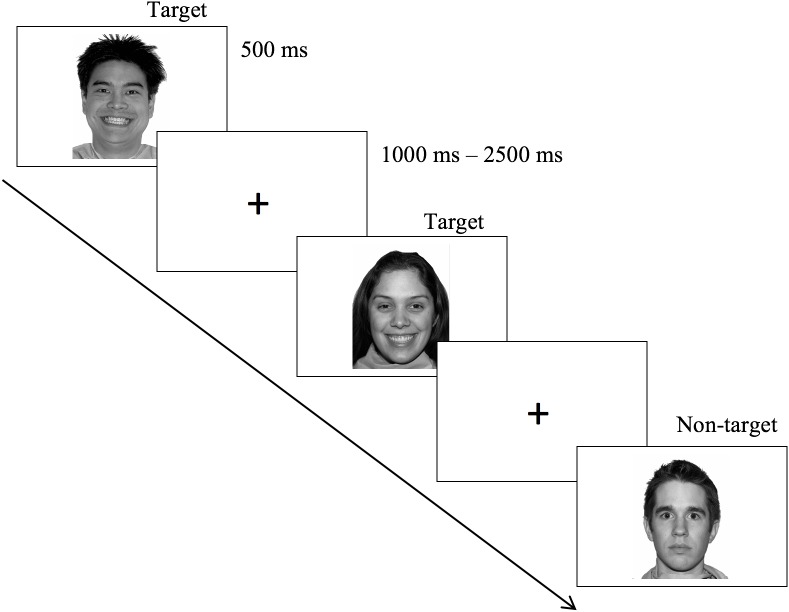
Schematic of emotional Go/No-go task. Example happy/neutral block. Target = “Go” stimulus, Non-target = “No-go” stimulus. Each block contained 35 targets and 13 non-targets. Stimuli are from the NimStim set of facial expressions (Tottenham et al., 2009). Actors provided written informed consent for the publication of these images. No copyright permissions are required for the use of these images.

Participants completed a Go/No-Go task employing the stimuli described above (see Figure 1). Task design was very similar to that employed by Hare et al. (2005, 2008) and Tottenham et al. (2011). At the start of each task block, participants read a screen stating the type of facial expression (e.g., Happy) that served as the target for that block. Participants were asked to press a button on a laptop computer keyboard for each target facial expression presentation (i.e., “Go” trials) and withhold response to any trials with any other type of facial expression (i.e., “No-Go” trials). Each type of facial expression served as target and non-target, in turn, across blocks, for 6 blocks of Target/Non-Target pairs: Fear/Neutral, Neutral/Fear, Happy/Fear, Fear/Happy, Happy/Neutral, Neutral/Happy. There was a nearly 3:1 ratio of “Go” to “No-Go” trials (35 targets and 13 non-targets per block) to develop a prepotent tendency to respond. Targets and non-targets were each shown for 500 ms and interspersed with a variable duration inter-stimulus fixation cross of 1–2.5 s to reduce impact of anticipatory effects. Each block took about 2.5 min to complete, and the sequence of blocks was varied between participants in a pseudo-random order to assure that two blocks with the same instruction (i.e., which type of facial expression they should respond to) were not adjacent (e.g., Neutral/Happy would not be immediately followed by Neutral/Fear). The start of each block was self-paced so participants could take a break between blocks if needed. However, nearly all participants completed the task without a break period. The experimental task took about 15 min to complete. The task was programmed in E-Prime 2.0 (Psychology Software Tools, Pittsburgh, PA) and completed on an HP ProBook laptop with 15.6 inch LED HD display.

### Data Analysis Plan

The primary aim for the present study was to identify the differential impact of individual emotions as targets and non-targets on younger and older adults’ task performance, so analyses focused upon blocks pairing neutral with emotional stimuli. Thus, the 4 blocks of Target/Non-Target (i.e., Go/No-go) pairs analyzed were: Fear/Neutral, Happy/Neutral, Neutral/Fear, and Neutral/Happy. The outcome measures computed for each individual in each task block were target miss rate, non-target false alarm rate, and response times to targets. To measure misses, we computed the proportion of targets where no response was provided (i.e., for “go” trials) out of the total number of targets. Higher miss rates reflect poorer emotion discrimination (i.e., button press incorrectly withheld because target facial expression was conflated with the non-target facial expression. To measure response inhibition, we computed false alarm rate as a proportion of non-targets where a response was provided (i.e., for “no-go” trials) out of the total number of non-targets. Response inhibition to neutral non-targets is an index of cognitive control, while response inhibition to emotional non-targets is an index of emotion regulation. Higher false alarm rates reflect poorer inhibitory control. To measure speed of responding, we computed mean of response times to targets where a response was provided, by block.

Three parallel 2 × 2 × 2 repeated measures ANOVAs were computed, one for each of the dependent outcome measures: missed responses to targets, false alarms to non-targets, and response times to targets. The ANOVAs assessed responses in the four task blocks of interest (Fear/Neutral, Happy/Neutral, Neutral/Fear, Neutral/Happy), with within-subjects factors of emotion (fear, happy) and stimulus type (emotion as target, emotion as non-target) and between subjects factor of age group (younger adults, older adults). Follow-up *t*-tests clarified the nature of interactive effects.

Exploratory analyses tested the possibility of a speed/accuracy trade-off in task performance, i.e., whether younger and older adults differentially prioritized speed or accuracy when responding (Salthouse, 1979; Schulz et al., 2007; Tottenham et al., 2011). To do so, the correlation between false alarms rates to non-targets and response times to targets was computed separately for each task block, by age group. Fisher’s *Z* tests then compared the strength of the correlation (i.e., speed/accuracy trade-off) between younger and older adults within each task block. Additionally, to more fully characterize the sample, we contrasted younger and older adults’ responses on neuropsychological measures of cognition and self-report measures using one-way ANOVAs. Exploratory analyses assessed the partial-correlation between response inhibition (false alarm rates) and cognitive and self-report measures, corrected for age. We applied Bonferroni correction when interpreting outcomes of correlational analyses to protect against problem of multiple comparisons (i.e., Type 1 error). All analyses were conducted using SPSS Version 25.0 (IBM SPSS Statistics, Armonk, NY).

## Results

### Neuropsychological Measures

Participants completed several neuropsychological measures to more fully characterize the sample and permit exploratory analyses of the relationship between task results of response inhibition and standardized assessments. Results of between-group comparisons of neuropsychological measures of cognition and self-report measures are available in Table 1. In summary, younger adults performed significantly better than older adults on all measures of cognition (i.e., faster responses, more words produced) with exception of Verbal Fluency to letters, and reported higher STAI state and trait anxiety levels. Older adults reported greater use of emotional reappraisal strategies in the ERQ-reappraisal subscale, but there were no group differences in use of emotional suppression. Exploratory analyses of partial-correlations between task results (false alarms to non-targets, by block; 4 outcome measures) and cognitive and self-report measures (16 outcome measures, as described in “Materials and Methods” section), corrected for age, did not surpass significance threshold for multiple comparisons (Bonferroni corrected threshold *p* < 0.0008 observed all *r*s < | 0.29|, all *p*s ≥ 0.01), indicating that Go/No-go task performance did not have relationship to neuropsychological or self-report measures. (Correlation results are reported in Supplementary Table S1).

### Ability to Discriminate Emotions: Missed Responses to Targets

**FIGURE 2:**
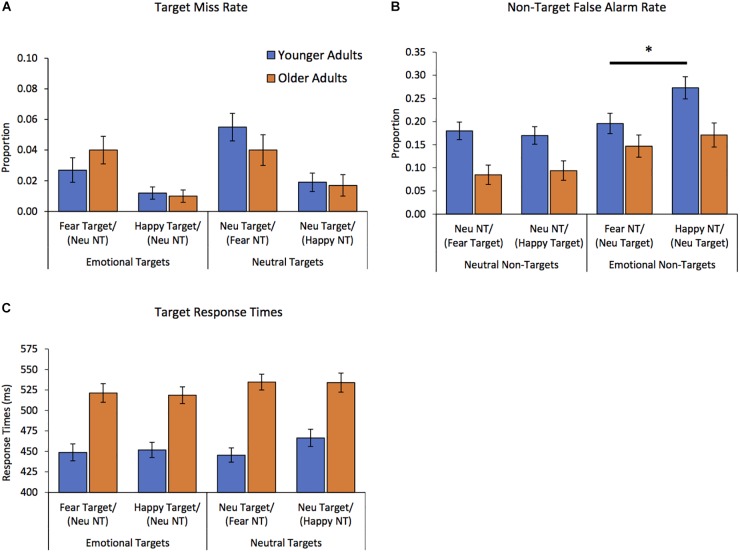
Results of Go/No-go task by outcome measure and age group. Each block contained 35 targets and 13 non-targets. **(A)** Target miss rate. There were more misses for neutral targets than emotional targets, and more misses in blocks with fearful faces than happy faces. There were no effects of age on target miss rate. **(B)** Non-target false alarm rate. Younger adults had more false alarms than older adults. There were more false alarms to happy than fearful non-targets, but false alarms to neutral non-targets did not differ between blocks with fearful vs. happy target faces. Interactive effects revealed only younger adults had significantly greater false alarms to happy than fearful non-target faces (denoted with black bar). ^∗^*p* < 0.0005, *d* = 0.59. **(C)** Target response times. Responses were faster to emotional targets than neutral targets, and younger adults were faster to respond to targets than were older adults. There were no differences between response times within blocks containing happy vs. fearful faces. Target = “Go” stimulus. NT = non-target “No-go” stimulus. Neu = neutral. Error bars represent standard error of the mean (SEM).

Results of the ANOVA on proportion of missed targets (as described in “Data Analysis Plan” section), showed two main effects. There was a main effect of stimulus type [*F*(1,78) = 5.67, *p* = 0.02, $\eta_{p}^{2}$ = 0.07] reflecting that on average there were fewer misses for emotional targets than for neutral targets (emotional targets *M* = 0.02, *SD* = 0.04; neutral targets *M* = 0.03, *SD* = 0.05). There was also a main effect of emotion [*F*(1,78) = 62.58, *p* < 0.0005, $\eta_{p}^{2}$ = 0.28] due to more misses in blocks with fearful faces than happy faces (fear *M* = 0.04, *SD* = 0.06, happy *M* = 0.01, *SD* = 0.03). One-sample *t*-tests confirmed that the proportion of misses in all blocks was greater than zero [*t*s(79) > 4.00, *p*s < 0.0005, *d*s > 0.45], confirming results were not subject to floor effects. Main effects are illustrated in Figure 2A. Notably, there were no effects of age on target miss rate; neither the main effect of age nor any interactions surpassed threshold for significant effects [all *F*s(1,78) < 2.59, *p*s > 0.11, $\eta_{p}^{2}$ < 0.033].

### Response Inhibition: False Alarms to Non-targets

Results of the ANOVA on proportion of false alarms to non-targets (as described in “Data Analysis Plan” section) revealed a more complex pattern of results. There was a main effect of age [*F*(1,78) = 9.36, *p* = 0.003, $\eta_{p}^{2}$ = 0.11] reflecting that younger adults had more false alarms than older adults (younger adults *M* = 0.20, *SD* = 0.15; older adults *M* = 0.12, *SD* = 0.12). There were also main effects of stimulus type [*F*(1,78) = 36.39, *p* < 0.0005, $\eta_{p}^{2}$ = 0.32] and of emotion [*F*(1,78) = 5.98, *p* = 0.017, $\eta_{p}^{2}$ = 0.07]. These main effects were qualified by an interaction between stimulus type and emotion [*F*(1,78) = 7.50, *p* < 0.01, $\eta_{p}^{2}$ = 0.09; See Figure 2B]. Follow up tests were conducted to clarify the nature of the interaction between stimulus type and emotion. Paired samples *t*-tests revealed a higher proportion of false alarms to happy than fearful non-targets [i.e., Neutral/Happy > Neutral/Fear; *t*(79) = 3.60, *p* = 0.001, *d* = 0.40; happy non-targets *M* = 0.23, *SD* = 0.17; fearful non-targets *M* = 0.17, *SD* = 0.15], but the proportion of false alarms to neutral non-targets did not differ between blocks with fearful vs. happy target faces [i.e., Fear/Neutral vs. Happy/Neutral; *t*(79) < 1, *M*s = 0.14, *SD*s = 0.13]. There was also a strong trend toward interaction among factors stimulus type, emotion, and age [*F*(1,78) = 3.76, *p* = 0.056, $\eta_{p}^{2}$ = 0.05].

In consideration of the strongly trending 3-way interaction among emotion, stimulus type, and age, we more closely explored how emotion impacted false alarm rates for younger and older adults. Separate *post hoc* paired-samples *t*-tests within each age group comparing their false alarms to happy vs. fearful non-targets (Neutral/Happy, Neutral/Fear) showed younger adults had significantly more false alarms to happy than fearful non-targets [i.e., Neutral/Happy > Neutral/Fear; *t*(43) = 3.79, *p* < 0.0005, *d* = 0.59; effects depicted in Figure 2B]. In contrast, older adults’ false alarm rates did not differ between happy and fearful non-targets [*t*(35) = 1.15, *p* = 0.26, *d* = 0.20]. Results of parallel paired-samples *t*-tests within each age group on their false alarms to neutral non-targets (Happy/Neutral, Fear/Neutral) reflected that neither older nor younger adult groups differed in their false alarm rates to neutral non-targets among happy vs. fearful targets (*t*s < 1, *p*s > 0.57, *d*s < 0.08). Thus, *post hoc* tests indicated that the strong 3-way trend was driven singularly by younger adults’ elevated false alarm rate to happy (vs. fearful) non-targets.

### Response Times to Targets

Results of the ANOVA on response times to targets (as described in “Data Analysis Plan” section), showed two main effects. There was a main effect of stimulus type [*F*(1,78) = 10.10, *p* = 0.002, $\eta_{p}^{2}$ = 0.12], indicating that participants responded faster to emotional targets than neutral targets (emotion *M* = 485.13, *SD* = 64.74; neutral *M* = 495.13, *SD* = 63.09). There also was a main effect of age [*F*(1,78) = 33.54, *p* < 0.0005, $\eta_{p}^{2}$ = 0.30], indicating that younger adults’ responses were faster than older adults’ responses (younger adults *M* = 453.18, *SD* = 64.24; older adults *M* = 527.08, *SD* = 65.59). Main effects depicted in Figure 2C. The main effect of emotion and all interactions did not reach significance [*F*s(1,48) < 2.14, *p*s > 0.14, $\eta_{p}^{2}$ < 0.028].

### Speed/Accuracy Trade-Off

Results of exploratory analyses of the speed/accuracy trade-off for each block showed a significant correlation between non-target false alarm rate and target response time for younger adults in the Neutral/Fear [r(44) = −0.44, *p* = 0.003] and Fear/Neutral blocks [r(44) = −0.53, *p* < 0.0005] and for older adults in the Neutral/Fear block [OA r(36) = −0.48, *p* = 0.003; Bonferroni corrected threshold for 8 tests, *p* < 0.006]. In each case faster response times corresponded with higher false alarm rate. (Full correlation results reported in Table 2). However, Fisher-*Z* tests of the strength of the speed/accuracy trade-off in each block between younger vs. older adults indicated there were no significant group differences (all *p*> 0.09); the trade-offs were in the same direction for both age groups and did not significantly differ in strength.

**Table 2:** Speed/accuracy trade-off.

Correlations between target response time and non-target false alarm rate					

			Block		
		Happy target/Neutral non-target	Fear target/Neutral non-target	Neutral target/Happy non-target	Neutral target/Fear non-target
Younger Adults	*r*	−0.36	−0.53	−0.36	−0.44
	*p*	0.02	<0.0005^*^	0.02	0.003^*^
Older Adults	*r*	−0.24	−0.20	−0.37	−0.48
	*p*	0.16	0.26	0.03	0.003^*^

*Younger adults n = 44, Older adults n = 36. ^∗^Significant after Bonferroni correction for multiple comparisons (p < 0.006).*

## Discussion

This study compared healthy older and young adults’ response inhibition to emotional faces. Our key finding was that although younger adults were faster to respond, older adults had better emotional response inhibition. This outcome provided partial support for the hypothesized age differences in emotional vs. non-emotional response inhibition, where we expected that older adults would show relative disadvantage for resisting neutral non-targets, but relative advantage for resisting emotional non-targets, when compared to younger adults. Results also fully supported the second hypothesis, that younger and older adults would both demonstrate response patterns aligning with approach/avoidance motivational goals; happy faces produced more approach-related behavior, while fearful faces produced more avoidance-related behavior. This was indexed by greater false alarms to happy than fearful non-targets, faster responses to happy than fearful targets, and fewer misses for happy than fearful targets. Importantly, we demonstrate for the first time that older adults have better emotional response inhibition than younger adults. Complementing previous literature in the domains of attention and memory, these results provide new evidence that in the domain of response inhibition older adults are able to employ greater emotion regulatory control than younger adults.

### Emotion Discrimination

Several empirical studies and reviews have concluded that the ability to discriminate basic emotions is relatively unchanged in late life (Kryla-Lighthall and Mather, 2009; Ebner et al., 2012), while others have found evidence of declining discrimination between negative facial expressions (but not positive Gunning-Dixon et al., 2003; Bailey et al., 2009). It is important to point out aging may differently impact emotion discrimination vs. emotion labeling (see also identification, recognition). Older adults generally decline in emotion labeling accuracy with age (Calder et al., 2003; Ruffman et al., 2008), but results are less consistent for effects of age on emotion discrimination. Older adults’ sustained ability to successfully discriminate emotional vs. neutral stimuli is likely supported by limbic responses (amygdala, medial prefrontal, anterior cingulate, basal ganglia, etc.) to emotional information that are sufficiently preserved even well into late life (Grieve et al., 2005; Williams et al., 2006; Ebner et al., 2012).

The present findings concur with evidence that older adults do not experience appreciable declines in emotion discrimination ability for happy or fearful faces, at least in the context of a response inhibition task. Across both the younger and older adult groups, on average, participants had greater ability to discriminate (i.e., differentiate) between facial expressions in blocks with emotional targets than neutral targets, as indexed by fewer misses for emotional than neutral targets. Participants had greater tendency to interpret neutral target faces as expressing emotion (both within blocks with fearful or happy face non-targets), than to interpret emotional expressions as neutral. There was also greater discrimination (i.e., fewer misses) in blocks containing happy than fearful faces [irrespective of whether the happy face appeared as the target (“go”) expression or non-targets (“no go”) expression]. However, the effects of stimulus type and emotion were distinct and did not interact. In other words, both younger and older adults had more difficulty discriminating neutral targets among emotional non-targets within a block than the reverse, and they were also poorer at discriminating between neutral and negative faces than between neutral and positive faces (see Figure 2A).

Results broadly support theories of approach and avoidance motivations, where individuals are more inclined to engage with positive stimuli (and are more quick to do so), relative to negative stimuli (reviewed by Elliot, 2006). Among blocks with emotional targets, both young and older adults had fewer misses in blocks containing positive than negative targets. This is a manifestation of approach-motivation, as also reported in previous research with adolescents and younger adults who completed an emotional Go/No-go task (Tottenham et al., 2011). Here we showed the same pattern of results is also evident in older adults.

### Cognitive Control

As introduced earlier, age differences in cognitive control abilities were evident in the pattern of results for inhibition of responses to non-targets. The proportion of false alarms to neutral non-targets was our measure of cognitive (i.e., non-affective) control over responses. Counter to expectations, younger adults demonstrated significantly poorer cognitive control than older adults, as indicated by their higher mean false alarm rate across all task blocks. These findings further build upon a substantial literature showing that teenage years and “emerging adulthood” (Arnett, 2000) are periods of significant prefrontal brain development, enabling increased control over impulsivity and automatic reactions (Todd et al., 2012; Cohen-Gilbert et al., 2014; Cohen et al., 2016).

One reason older adults had fewer false alarms than younger adults may be because the task instructions asked participants to overtly evaluate the emotionality of the facial expressions trial-by-trial, thereby playing to the strengths of older adults; namely, engagement of chronically active emotion regulatory processes (reviewed by Kryla-Lighthall and Mather, 2009; Mather, 2012), even in blocks with neutral faces as targets. It is also possible that older adults may have been processing the present task’s facial stimuli (regardless of block) in a more self-relevant fashion than the stimuli used in studies that observed declining cognitive control with aging (e.g., Stroop tasks, inhibition tasks with symbols or shapes). As such, affective and self-relevant elements of the present task would be likely to have engaged medial prefrontal brain regions (Northoff et al., 2006; Gutchess et al., 2010; van Reekum et al., 2018), which are relatively well preserved in the aging process, in contrast to lateral prefrontal regions, which are engaged to a greater extent in cognitive tasks without self-relevant aspects (Raz et al., 2004; Grieve et al., 2005). These several possibilities are not mutually exclusive. However, in the absence of neuroimaging data in the present study, they are speculative possibilities that remain to be empirically tested.

### Emotion Regulation

Across younger and older participant groups, control over responses to neutral non-targets differed from responses to emotional non-targets, evidencing differential impact of emotion on regulation of responses. There was a strong effect of stimulus type, evident in the elevated false alarm rates observed when emotional faces (vs. neutral) served as non-targets. In particular, there were more false alarms to happy than fearful face non-targets, in keeping with prior literature in younger adults indicating general approach-motivation related responding [i.e., greater engagement with happy faces than negative faces (Hare et al., 2005; Phaf et al., 2014)]. It is also worth highlighting that false alarm rates for neutral non-targets did not differ as a function of whether they appeared among happy compared to fearful target faces (i.e., within Happy/Neutral vs Fear/Neutral blocks), suggesting that the block’s *target* emotion did not differentially lead to carry-over effects to the false alarm rate, and that in fact the false alarm response patterns were specifically a response to the non-target face’s emotionality (i.e., within Neutral/Happy, Neutral/Fear blocks).

Although there were no significant interactions with age for false alarm rates, results trended toward 3-way interaction among emotion, stimulus type, and age (*p* = 0.056) with a small to medium effect size. Across all of the outcome measures in this study, this is the only indication of interactive effects of age on emotional response inhibition. Follow-up analyses to elucidate the trending interaction revealed that it was driven particularly by younger adults’ significantly higher false alarm rates to positive face non-targets (compared to negative; Schulz et al., 2007), whereas older adults responded similarly to positive and negative face non-targets. The age by emotion interaction for false alarms was limited to emotional non-targets; there was no interaction here between age and emotion in blocks with neutral non-targets (Schel and Crone, 2013). Notably, the 3-way interaction among emotion, stimulus type, and age reached a more stringent level of significance (results reported in Supplementary Materials) after consideration of the influence of age differences in anxiety (Machado et al., 2018), which can increase attention and false alarm rates to negative information in particular (Mogg and Bradley, 2005; Pacheco-Unguetti et al., 2012). This exploratory finding confirms that the interactive effects of age were not attributable merely to differing degrees of reported anxiety between younger and older adults. Results suggest that age may particularly impact response inhibition to positive non-targets, with older adults demonstrating relatively better regulation of responses than younger adults.

Taken together with higher miss rate for fearful than happy face targets, results of higher false alarms for happy than fearful face non-targets align with a substantial literature suggesting that there is an overall greater approach motivation to engage with positive than negative information. Another, and complementary, interpretation is that happy faces were more disruptive to response patterns (than fearful faces) because there was a lesser degree of chronically active emotion regulatory processes engaged to inhibit responses to happy faces. It is possible that the lower false alarm rates to fearful faces were a result of ongoing regulatory control to not engage with negative faces, whereas happy faces were not subject to such ongoing automatic regulation. If this is the case, it is also worth considering why we did not observe significant interactive effects of age on false alarms to emotional non-targets (i.e., age × stimulus type). A substantial literature posits that older adults may have stronger ongoing emotion regulatory control processes to maximize positive and minimize negative affect, compared to younger adults (reviewed by Kryla-Lighthall and Mather, 2009; Mather, 2012). As such, older adults could be expected to also have strong approach motivation toward positive information, and consequently higher false alarm rates for positive vs. negative information. However, as observed in follow-up analyses, happy and fearful face non-targets produced comparable false alarm rates in older adults. One likely reason that older adults had comparable false alarm rates for positive and negative non-targets could be that the Go/No-go task requires a high degree of cognitive resources to respond and inhibit responses correctly. If older adults were dedicating their full effort and attention to the task, then they may not have had sufficient remaining cognitive resources available for automatic emotion regulatory strategies to be reflected in task performance (Kryla-Lighthall and Mather, 2009; Samanez-Larkin et al., 2009; Wegner, 1994). This interpretation is supported by results of several previous studies and a meta-analysis reporting less evidence of automatic emotion regulation processes under conditions of high cognitive demand (Mather and Knight, 2005; Knight et al., 2007; Reed et al., 2014), and provides further evidence that the age-related positivity bias is less apparent in tasks requiring greater cognitive resources. In summary, results suggest that in the context of task instructions to avoid responding to emotional expressions (i.e., happy, fearful non-targets), older adults are better able to regulate their responses than younger adults. The present findings extend the trajectory of results reported in younger adults (Tottenham et al., 2011; Schel and Crone, 2013) to show continued development of regulation over responses to emotional information from younger to older adulthood.

It is also worth noting that although response times were faster to emotional than neutral targets, the emotion (i.e., positive vs. negative expression) of the face did not influence speed of responses. There was a general faciliatory effect of emotion on response times to targets, which extended to younger and older adults. Results support a general faciliatory effects of emotion on response times to targets (Chiu et al., 2008), and also may reflect that inhibition of neutral non-targets (i.e., in emotional target blocks) is less cognitively demanding (i.e., consequently faster processing) than inhibition of emotional non-targets (Zinchenko et al., 2017). Taken together, although participants made more false alarms to positive than negative non-targets, emotion had a broadly faciliatory effect on response times to emotional targets (vs. neutral), which did not differ between positive and negative facial expressions.

### Speed/Accuracy Trade-Off

To rule out the possibility that results were merely reflective of an age-related difference in “calculation” of a speed/accuracy trade-off between response times to targets and false alarm rate (Salthouse, 1979; Schulz et al., 2007), we assessed the strength of the trade-off for each block and contrasted younger and older adults. Although older adults were slower to respond than younger adults overall, speed/accuracy correlation results showed that younger and older adults both demonstrated a trade-off favoring speed at the expense of accuracy, most robustly within blocks with negative faces. Critically, there were no significant differences between the strength of the speed/accuracy trade-off between younger and older adults. This finding confirms that conclusions of better emotional response regulation in older adults is not merely due to older adults differentially resolving the speed/accuracy trade-off in favor of accuracy while younger adults resolve in favor of response speed.

### Limitations and Future Directions

There were a few limitations to our study design that present interesting opportunities for future investigations. Our design did not include blocks featuring neutral faces both as the targets and non-targets, i.e., employing instructions that depend on a dimension of the stimuli other than facial expression. A comparative task condition with no overt emotional stimuli and without affectively focused instructions could provide a more “pure” reflection of cognitive control over non-emotional information (Smittenaar et al., 2015) to allow comparison to emotional response inhibition. Future studies may also wish to employ videos of dynamic facial expressions (Zinchenko et al., 2017) or a wider range of emotions, such as sadness or anger to better probe not only valence differences (positive/negative), but also expand the range of stimulus arousal level (high/low) effects on response inhibition. We did not collect normative ratings of valence or arousal from participants so we were not able to evaluate task responses as a function of self-reported individual responses to facial expressions. Collecting individual participants’ interpretation of stimuli could allow a dimensional approach to analyses rather than categorical understanding of facial expression discrimination and response inhibition.

The present study extended existing literature of emotional response inhibition beyond younger adults and into late life, however, we did not include participants in the middle of the lifespan. Thus, it remains to be determined whether there is a linear increase in emotional response inhibition throughout the lifespan, or instead whether there is a peak in midlife that falls again somewhat in later life (or even other non-linear trajectories). It would also be illuminating to understand how symptoms of late-life depression and anxiety or pathological aging conditions like Alzheimer’s disease could influence the pattern of results of emotional inhibition. It is possible that the atrophy or pathology differentially affecting some areas of the brain to greater extents may lead to not only global decreases in performance, but also fundamentally different patterns of responses to positive vs. negative stimuli (Waring et al., 2017), when compared to aging individuals without clinically significant atrophy or Alzheimer pathology. Future research employing neuroimaging methodology during task performance in healthy and pathological aging will elucidate these possibilities.

## Conclusion

In summary, happy faces produced more approach-related behavior, while fearful faces produced comparatively more avoidance-related behavior, and this effect was seen in both younger and older adults. Although younger adults were faster to respond, older adults had better response inhibition than younger adults. Older adults’ enhanced adaptive emotion regulation strategies may facilitate resistance to emotional distraction, suggesting older adults more effectively employ emotion regulatory ability, although on a slower time course than younger adults. The current study fills a critical gap in the literature; although previous studies have investigated older adults’ ability to select among competing stimulus attributes (e.g., emotion conflict or emotional Stroop tasks), there are no published reports of older adults’ ability to stop (inhibit) their responses to emotional information. Despite the lack of empirical research attention garnered, emotional response inhibition in late life is an important topic, with implications for better understanding how older adults engage, or inhibit engagement, with aversive and pleasant information. One’s degree of engagement with emotional stimuli also has downstream effects on subsequent ability to regulate thoughts and behavioral responses to emotional information (Gross, 2001; Urry and Gross, 2010); the greater the initial engagement, the greater the affective response (i.e., autonomic response and limbic reactivity) requiring regulation. As younger and older adults often employ different cognitive strategies and have differing affective goals (Reed and Carstensen, 2012; Urry and Gross, 2010), elucidation of age-specific response patterns are highly relevant toward developing more precise models of cognitive aging and age-appropriate focused behavioral interventions for maximizing positive affect and minimizing negative affect in late life. The present study extends the literature of emotional response inhibition in younger adulthood into late life, and in doing so further illuminates the important ways in which cognitive aging interacts with affective control processes.

## Ethics Statement

The protocol was approved by the Institutional Review Boards of Saint Louis University, Santa Clara University, and Washington University in St Louis. All participants provided written informed consent in accordance with the Declaration of Helsinki. Participants at Saint Louis University and Washington University School of Medicine additionally provided HIPAA authorization.

## Author Contributions

JW conceived and designed the study and conducted the data analyses. JW and TG collected the data. JW, TG, and EL drafted and revised the manuscript. All authors granted approval for publication of the content.

## Conflict of Interest Statement

The authors declare that the research was conducted in the absence of any commercial or financial relationships that could be construed as a potential conflict of interest.

## Footnotes

1 https://danlab7.wixsite.com/nimstim
