# Supplementary material for: Emotional Response Inhibition Is Greater in Older Than Younger Adults
Source: Front Psychol. 2019 May 1;10:961. doi: 10.3389/fpsyg.2019.00961 (PMC6504835; doi:10.3389/fpsyg.2019.00961)
Supplement: Supplementary file 1 [file Data_Sheet_1.PDF]

Supplemental Materials

Emotional response inhibition is greater in older than younger adults

Jill D. Waring, Taylor R. Greif, & Eric J. Lenze

Methods

Participant Sample

Of the final sample of 44 younger adults, 20 participated at Saint Louis University and 24 participated at Santa Clara University. Of the final set of 36 older adults, 27 participated at Saint Louis University and 9 participated at Washington University in St Louis.

The participant sample was largely Caucasian and non-Hispanic. The race and ethnicity demographics counts among the younger adult sample were: 14 Asian-American, 3 Black or African-American, and 27 Caucasian, with 2 Hispanic participants. The race and ethnicity demographics counts among the older adult sample were: 2 Asian-American, 4 Black or African-American, and 30 Caucasian participants.

Stimuli

The NimStim (Tottenham et al., 2009) identities of the happy, neutral, and fearful faces used were numbers: 6, 8, 11, 14, 15, 16, 27, 28, 36, 39, 43, and 45. The set comprised 2 female African-American models, 2 female Asian models, 2 female Caucasian models, 2 male African-American models, 3 Caucasian male models, and 1 Asian male model. Ratings of validity and reliability are reported by Hare & colleagues (2008, supplementary Tables 2a and 4). Each model identity was viewed as both target and non-target within each block (i.e., with differing expressions), but model identities were not repeated back-to-back (i.e., irrespective of target or non-target status). All models identities were shown in each block. Target identities were repeated 2-3 times within each block.

Missing data on cognitive and self-report measures

Some measures were not administered during protocol development and pilot testing. Trail Making Test was not administered to 2 younger adult 4 older adult pilot participants. GDS and ERQ each were not administered to 1 older adult pilot participant.

There were missing data for the DKEFS Color Word Interference task due to participant and researcher error. The inhibition/switching condition was not administered to participants at Santa Clara University due to experimenter error. One younger adult participant spoke too rapidly to be intelligible in the Color naming and Word reading conditions. One older adult repeated a portion of the Inhibition condition, unbeknownst to researcher, so responses were not scored reliably. Completion time for two older adult participants was not recorded for the Inhibition/Switching condition due to experimenter error.

One older and one younger adult participant made an error on Trail Making Test Part B that was not detected by researcher until completion of the measure, rendering recorded time invalid.

Results

Table S1: Partial correlations between task performance and measures, controlling for age

		False alarm rate to non-targets			
		Neutral NT (happy Tar)	Neutral NT (fear Tar)	Happy NT (neu Tar)	Fear NT (neu Tar)
D-KEFS Color Word Interference					
Color naming (sec)	*r*	0.11	0.07	0.21	0.03
	p	0.34	0.52	0.06	0.80
	df	76	76	76	76
Word reading (sec)	*r*	0.03	-0.02	0.11	-0.09
	p	0.78	0.85	0.35	0.43
	df	76	76	76	76
Inhibition (sec)	*r*	0.29	0.18	0.21	0.19
	p	0.01	0.12	0.07	0.10
	df	76	76	76	76
Inhibition/switching (sec)	*r*	0.04	-0.02	-0.04	-0.05
	p	0.80	0.90	0.80	0.70
	df	51	51	51	51
D-KEFS Verbal Fluency					
Letter fluency: FAS	*r*	-0.02	-0.16	0.11	0.07
	p	0.85	0.16	0.36	0.56
	df	77	77	77	77
Category fluency: Animals + Boys' Names	*r*	0.07	0.02	0.17	0.05
	p	0.52	0.88	0.15	0.69
	df	77	77	77	77
Category Switching: Fruits/Furniture	*r*	0.04	0.07	0.00	-0.10
	p	0.76	0.55	0.98	0.38
	df	77	77	77	77
Trail Making Test					
Part A (sec)	*r*	0.20	-0.10	0.12	0.04
	p	0.10	0.42	0.33	0.73
	df	71	71	71	71
Part B (sec)	*r*	0.18	0.15	0.04	0.20
	p	0.14	0.21	0.76	0.09
	df	69	69	69	69
ERQ reappraisal (avg)	*r*	-0.10	-0.03	-0.14	0.03
	p	0.38	0.83	0.21	0.82
	df	76	76	76	76
ERQ suppression (avg)	*r*	-0.08	0.07	0.08	-0.08
	p	0.49	0.54	0.50	0.51
	df	76	76	76	76
STAI: state	*r*	0.05	0.12	0.09	0.23
	p	0.65	0.30	0.42	0.04
	df	77	77	77	77
STAI: trait	*r*	0.15	0.25	0.17	0.11
	p	0.19	0.03	0.14	0.34
	df	77	77	77	77
Beck Depression Inventory	*r*	0.15	0.17	0.17	0.19
	p	0.35	0.28	0.28	0.22
	df	41	41	41	41
Geriatric Depression Scale	*r*	0.22	0.33	0.06	0.02
	p	0.22	0.06	0.73	0.90
	df	32	32	32	32

Note: Citations available in text. None of the correlations surpassed threshold for significance after Bonferroni correction for multiple comparisons (p<.0008). BDI and GDS were only for one age group each. Color-word interference task was not collected on participants enrolled at Santa Clara University due to experimenter error. Neu=neutral face, Tar = target, NT=non-target

Effects of Anxiety on False Alarm Rates

After observing that the younger adults reported greater symptoms of anxiety than older adults (see main text Table 1), we explored the possibility that apparent age effects on false alarm rates were better explained by group differences in anxiety. Literature has demonstrated that anxiety impacts attention to emotional faces (Mogg & Bradley, 2005; Pacheco-Unguetti, Acosta, Lupiáñez, Román, & Derakshan, 2012). In particular, anxiety corresponds with higher false alarm rates to negative information. Thus, we repeated the omnibus ANOVA on false alarm rate including anxiety (STAI-state) as a covariate. STAI-state anxiety was examined (as opposed to STAI-trait anxiety) because it indexes present level of anxiety in the moment, and may be more reliable in older adults in particular because it does not rely on recollection of how one 'generally' feels. (STAI-state and STAI-trait were highly correlated [r=.61, p<.0005]). Results demonstrated that the trend toward 3-way interaction among factors of emotion, stimulus type, and age (reported in main text) reached a higher degree of significance when correcting for effect of anxiety ($F(1,77)=4.16$, $p<.05$, $\eta_p^2=.05$), with all effects in the same direction as the initial analyses of false alarm rates without anxiety as a covariate. This result confirms that the relationship between age and false alarm rate was not an artifact of age group differences in self-reported anxiety symptoms.

Task accuracy: d-prime

As a measure of overall accuracy in response discrimination, we computed d-prime, which is calculated from formulas for signal detection theory; d-prime= Z_{FA} - $Z_{H,}$ where Z_{FA} = Z score of the false alarms rate and Z_H = Z score of the hit rate (MacMillan & Creelman, 2005; Snodgrass & Corwin, 1988). D-prime values are undefined when the proportion of responses equals 0 or 1. Thus, to account for possible floor or ceiling values among false alarm or hit rates, respectively, values for d-prime were converted using the formulas from Snodgrass and Corwin (1988); Hits rate = (# hits + 0.5)/(# target trials + 1); False alarms rate = (#false alarms + 0.5)/(# non-target trials + 1). Higher d-prime values reflect better discrimination accuracy between facial expressions serving as targets versus non-targets, while zero represents chance performance. A 2x2x2 repeated measures ANOVA was computed on d-prime scores in the same fashion as for other task outcome measures; within-subjects factors of emotion (fear, happy) and stimulus type (emotion as target, emotion as non-target) and between subjects factor of age group (younger adults, older adults).

Results of the ANOVA on d-prime scores showed three main effects. There was a main effect of stimulus type ($F(1,78)=50.21$, $p<.0005$, $\eta_p^2=.39$), evidencing greater emotion discrimination for blocks with emotional targets than neutral targets (emotional target M= 3.09, SD=.68); neutral target M= 2.74, SD=.70). There was a main effect of emotion ($F(1,78)=10.37$, $p<.002$, $\eta_p^2=.12$), due to greater emotion discrimination in blocks with happy than fear faces (Fearful M= 2.83, SD=.70; Happy M=3.00, SD=.68). One-sample t-tests confirmed that emotion discrimination in all blocks was greater than zero, i.e., better than chance (ts(79)>33.06, ps<.0005, ds>3.71). Lastly, there was a main effect of age ($F(1,78)=8.01$, $p<.006$, $\eta_p^2=.09$); older adults had greater emotion discrimination than younger adults (younger adults M=2.73, SD=.60; older adults M=3.09, SD=.53). These main effects are illustrated in Figure S1. All interactions did not reach level of significance (Fs(1,78)<1.14, ps>.29, $\eta_p^2<.01$).

Figure S1: Go/No-go task response discrimination (d-prime) by outcome measure and age group

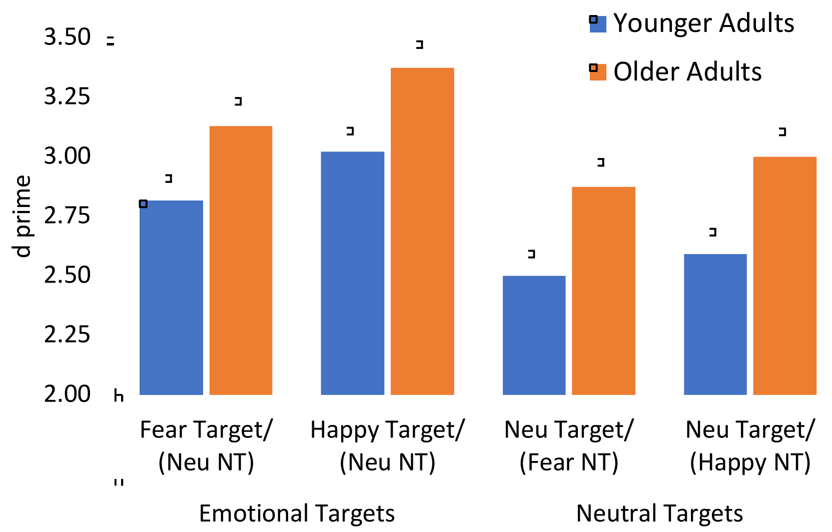

Notes: Each block contained 35 targets and 13 non-target distractors. Results showed greater emotion discrimination in blocks with emotional targets than neutral targets, greater emotion discrimination in blocks with happy than fear faces, and that older adults had greater response discrimination than younger adults. There were no significant interactions among factors. Legend: Target = 'Go' stimulus. NT= non-target 'No-go' stimulus. Neu= neutral. Error bars represent standard error of the mean (SEM).

References

Hare, T. A., Tottenham, N., Galvan, A., Voss, H. U., Glover, G. H., & Casey, B. J. (2008). Biological Substrates of Emotional Reactivity and Regulation in Adolescence During an Emotional Go-Nogo Task. *Biological Psychiatry*, *63*(10), 927–934. https://doi.org/10.1016/j.biopsych.2008.03.015

MacMillan, N. A., & Creelman, C. D. (2005). *Detection theory: A user's guide*. New Jersey: Lawrence Erlbaum.

Mogg, K., & Bradley, B. P. (2005). Attentional Bias in Generalized Anxiety Disorder Versus Depressive Disorder. *Cognitive Therapy and Research*, *29*(1), 29–45. https://doi.org/10.1007/s10608-005-1646-y

Pacheco-Unguetti, A., Acosta, A., Lupiáñez, J., Román, N., & Derakshan, N. (2012). Response inhibition and attentional control in anxiety. *Quarterly Journal of Experimental Psychology*, *65*(4), 646–660. https://doi.org/10.1080/17470218.2011.637114

Snodgrass, J., & Corwin, J. (1988). Pragmatics of measuring recognition memory-Applications to dementia and amnesia. *Journal of Experimental Psychology: General*, *117*(1), 34–50.

Tottenham, N., Tanaka, J. W., Leon, A. C., McCarry, T., Nurse, M., Hare, T. A., … Nelson, C. (2009). The NimStim set of facial expressions: Judgments from untrained research participants. *Psychiatry Research*, *168*(3), 242–249. https://doi.org/10.1016/j.psychres.2008.05.006
